# Supplementary material for: Combining density functional theory with macroscopic QED for quantum light-matter interactions in 2D materials
Source: Nat Commun. 2021 May 13;12:2778. doi: 10.1038/s41467-021-23012-3 (PMC8119442; doi:10.1038/s41467-021-23012-3)
Supplement: Supplementary file 1 — Supplementary information [file 41467_2021_23012_MOESM1_ESM.pdf]

Supplementary material: Combining density functional theory with macroscopic QED for quantum light-matter interactions in 2D materials

Mark Kamper Svendsen,[*,†,‖] Yaniv Kurman,[‡,‖] Peter Schmidt,[¶] Frank Koppens,[¶] Ido Kaminer,[‡] and Kristian S. Thygesen[§]

†*CAMD, Department of Physics, Technical University of Denmark, 2800 Kgs. Lyngby, Denmark*

‡*Department of Electrical Engineering, Technion, Israel Institute of Technology, 32000 Haifa, Israel*

¶*ICFO-Institut de Ciencies Fotoniques, The Barcelona Institute of Science and Technology, 08860 Castelldefels (Barcelona), Spain*

§*CAMD and Center for Nanostructured Graphene (CNG), Department of Physics, Technical University of Denmark, 2800 Kgs. Lyngby, Denmark*

‖*These authors contributed equally to the manuscript*

E-mail: *markas@dtu.dk

Contents

- Supplementary note 1: The MQED Wigner-Weisskopf model
- Supplementary note 2: Dipole moment scaling of rates
- Supplementary note 3: Reflection from a uniaxial material
- Supplementary note 4: Calculating the diamagnetic $\hat{\boldsymbol{A}}^{\mathbf{2}}$ term.
- Supplementary note 5: Lamb shift renormalization of the intersubband transition energy
- Supplementary note 6: Examples of time dependent excited state probabilities
- Supplementary note 7: Simulation details/Convergence

Supplementary note 1: The MQED Wigner-Weisskopf model

In this section we derive the extensions to the theory from Ref.[1] needed to describe the emission from the intersubband transitions in the presence of the optical environment as defined by Macroscopic quantum electrodynamics (MQED)[2] and the Dyadic Green's function (DGF).

We will use the Wigner Weisskopf model to examine the interaction of an emitter with the modes of a general magnetoelectric background. We consider the case of an emitter initially in the excited state at $t = 0$. This emitter can then decay into one of a series of ground states, via the continuum of modes as defined by the electromagnetic environment. The full time dependent state of the system is described by Eq.(4) in the main text. That expression can be understood as a specific form of the more general expression for the time dependent state of a system that at $t = 0$ is in an initial state i, and then decays into one of a set of final states $\{f\}$ via one of the modes of the electromagnetic field $\{\boldsymbol{M}\}$:

$$|\psi(t)\rangle = C_i(t)e^{-i\omega_i t}|i\rangle \otimes |0, ..., 0\rangle + \sum_{f,\mathbf{M}} C_{f,\mathbf{M}}(t)e^{-i(\omega_{\mathbf{M}}+\omega_f)t}|f\rangle \otimes |0, \ldots, 1_{\mathbf{M}}, \ldots, 0\rangle \,, \quad (1)$$

where $1_{\mathbf{M}}$ denotes one quantum of a specific electromagnetic excitation, $\mathbf{M}$, ω_i/ω_f denotes the electrons initial/final state frequencies, and $\omega_{\mathbf{M}}$ denotes the frequency of the electromagnetic excitation. $C_i(t)$ and $C_{f,\mathbf{M}}(t)$ describe the amplitudes of the electrons initial and final states. In the case of the van der Waals heterostructure considered in this work, the interpretation of the $\sum_{f,\boldsymbol{M}}$ is as a sum over modes of the electromagnetic environment and final states in the upper subband, as defined by their in-plane momentum $\boldsymbol{q}$ and the dispersion relation of the subband. Noticeably, Eq. (1) describes only a single quanta excitation which can be in the excited state, or as a coherent superposition between many final states and electromagnetic modes. Using this expression for the wavefunction neglects high-order processes such as

two-photon emission in addition to the counter-rotating terms. In the structure discussed in the paper, these contributions are weak and therefore neglected, though stronger interaction would force a wavefunction extension into additional terms.

Generally, the time dependent state of the system, Eq.(1), will evolve according to the time dependent Schrödinger equation. To determine its dynamics we write down the three term Hamiltonian of the coupled system:

$$H = H_{\mathrm{E}} + H_{\mathrm{F}} + H_{\mathrm{int}} \,. \tag{2}$$

The emitter Hamiltonian can be written as:

$$H_E = \hbar \sum_f \omega_f \hat{b^\dagger}_f \hat{b}_f + \hbar\omega_i \hat{b^\dagger}_i \hat{b}_i \,, \tag{3}$$

with $\hat{b}_{i,f}$ and $\hat{b}^\dagger_{i,f}$ representing the initial state annihilation and creation operators respectively, i, or final states, f, respectively. The field Hamiltonian can be expressed in two forms,

$$H_{\mathrm{F}} = \hbar \sum_{\mathbf{M}} \omega_{\mathbf{M}} \hat{a}^\dagger_{\mathbf{M}} \hat{a}_{\mathbf{M}} = \sum_{j=x,y,z} \int d\mathbf{r} d\omega \hbar\omega \hat{f}^\dagger_j(\mathbf{r},\omega) \hat{f}_j(\mathbf{r},\omega) \,, \tag{4}$$

symbolically as a sum over modes, $\boldsymbol{M}$, with operators $\hat{a}^\dagger_{\boldsymbol{M}}$ and $\hat{a}_{\boldsymbol{M}}$ for creating and annihilating each mode respectively, or using the MQED notations so that each mode is represented as a dipole creation and annihilation (using the operators $\hat{f}^\dagger_j(\mathbf{r},\omega)$ and $\hat{f}_j(\mathbf{r},\omega)$ respectively) located at $\boldsymbol{r}$, with spatial orientation $j = x, y, z$ and frequency ω. The symbolic sum over modes should thus be understood in the following way,

$$\sum_{\mathbf{M}} \rightarrow \int d\omega \int d^3r \sum_{j=x,y,z} \,. \tag{5}$$

As discussed in the main text, the MQED vector potential in the Weyl gauge is given

in terms of the dipole excitations of the combined light-matter system:[2]

$$\hat{A}_i(\mathbf{r}) = \sqrt{\frac{\hbar}{\pi\epsilon_0}} \int d\omega \frac{\omega}{c^2} \int d^3s\, \overleftrightarrow{G}_{il}(\mathbf{r},\mathbf{s},\omega) \sqrt{\mathrm{Im}\,\epsilon_{lj}(\mathbf{s},\omega)} \hat{f}_j(\mathbf{s},\omega) + \text{h.c.}\,, \tag{6}$$

Finally, the Weyl gauge minimal coupling Hamiltonian becomes,

$$\hat{H}_{\text{int}} = \frac{e}{m}\hat{\boldsymbol{A}}\cdot\hat{\boldsymbol{p}} - \frac{ie\hbar}{2m}\boldsymbol{\nabla}\cdot\hat{\boldsymbol{A}} = \sum_{f,M}\hbar g_{i,f\boldsymbol{M}} \hat{b^\dagger}_i \hat{b}_f \hat{a}_M + h.c.\,, \tag{7}$$

where on one hand we use the electron mass, m, electron charge, e, the electric vector potential, $\hat{\boldsymbol{A}}(\boldsymbol{r})$, and momentum operator $\hat{\boldsymbol{p}}$, and on the other hand the coupling constants, $g_{i,f\boldsymbol{M}}$, that couples the initial state with each optical excitation and allowed final state.

Inferring that the state has to obey the time dependent Schrodinger equation, the equations of motion for the state coefficients become:

$$\partial_t C_i(t) = -i\sum_{f,\mathbf{M}} g_{i,f\mathbf{M}} C_{f,\mathbf{M}}(t) e^{-i\omega_{if\boldsymbol{M}}t}\,, \tag{8}$$

$$\partial_t C_{f,\mathbf{M}}(t) = -i g^*_{i,f\mathbf{M}} C_i(t) e^{i\omega_{if\boldsymbol{M}}t}\,, \tag{9}$$

where we define $\omega_{if\boldsymbol{M}} = \omega_{\mathbf{M}} + \omega_f - \omega_i$. Using equations 8 and 9 leads to the closed expression for time evolution of the excited state coefficient:

$$\partial_t C_i(t) = -\sum_{f,\mathbf{M}} \int dt_1 |g_{i,f\mathbf{M}}|^2 e^{-i\omega_{if\boldsymbol{M}}(t-t_1)} C_i(t_1)\,. \tag{10}$$

In the following we will show that for a stratified medium like the vdW-heterostructure considered in this work, equation 10 can be written in terms of a interaction kernel, $K(\boldsymbol{q},\omega)$, in the following way:

$$\dot{C}_i(t) = -\int_0^t dt' \int d\omega \int d\boldsymbol{q} K(\omega,\boldsymbol{q}) e^{-i(\omega-\omega_{if\boldsymbol{q}})(t-t')} C_i(t')\,, \tag{11}$$

where $\hbar\omega_{if\mathbf{q}} = \varepsilon_{f\mathbf{q}} - \varepsilon_i$ is the dispersive intersubband transition energy. As discussed in the main text, the kernel $K(\boldsymbol{q},\omega)$ is a function of frequency, ω, and in-plane momentum, $\boldsymbol{q}$, and it holds all of the information about the coupling to the electromagnetic environment. The integral kernel is made up of three different contributions,

$$K(\boldsymbol{q},\omega) = K_{\hat{\boldsymbol{A}}\cdot\hat{\boldsymbol{p}}}(\boldsymbol{q},\omega) + K_{\nabla\cdot\hat{\boldsymbol{A}}}(\boldsymbol{q},\omega) + 2\mathrm{Re}[K_{cross}(\boldsymbol{q},\omega)]\,, \tag{12}$$

arising from the $\hat{\boldsymbol{A}}\cdot\hat{\boldsymbol{p}}$ and $\nabla\cdot\hat{\boldsymbol{A}}$ terms in the interaction Hamiltonian and their cross terms respectively. This follows from the expansion of the matrix elements in equation 10:

$$\begin{aligned}
&\hbar^2\sum_{\mathbf{M}}|g_{i,f\mathbf{M}}|^2 e^{-i\omega_{if\boldsymbol{M}}(t-t_1)} \\
&=\sum_f\int d\omega\int d^3s\sum_{j=x,y,z}\left|\langle f,\boldsymbol{s}\omega j|\frac{e}{m}\hat{\boldsymbol{A}}\cdot\hat{\boldsymbol{p}} - \frac{ie\hbar}{2m}\nabla\cdot\hat{\boldsymbol{A}}|i,0\rangle\right|^2 e^{-i(\omega-\omega_{if})(t-t_1)} = \\
&\frac{e^2}{m^2}\sum_f\int d\omega\int d^3s\sum_{j=x,y,z}\Big(\langle i,0|(\hat{\boldsymbol{A}}\cdot\hat{\boldsymbol{p}})^\dagger|f,\boldsymbol{s}\omega j\rangle\langle f,\boldsymbol{s}\omega j|\hat{\boldsymbol{A}}\cdot\hat{\boldsymbol{p}}|i,0\rangle + \\
&\frac{i\hbar}{2}\langle i,0|(\nabla\cdot\hat{\boldsymbol{A}})^\dagger|f,\boldsymbol{s}\omega j\rangle\langle f,\boldsymbol{s}\omega j|\hat{\boldsymbol{A}}\cdot\hat{\boldsymbol{p}}|i,0\rangle - \frac{i\hbar}{2}\langle i,0|(\hat{\boldsymbol{A}}\cdot\hat{\boldsymbol{p}})^\dagger|f,\boldsymbol{s}\omega j\rangle\langle f,\boldsymbol{s}\omega j|\nabla\cdot\hat{\boldsymbol{A}}|i,0\rangle \\
&\frac{\hbar^2}{4}\langle i,0|(\nabla\cdot\hat{\boldsymbol{A}})^\dagger|f,\boldsymbol{s}\omega j\rangle\langle f,\boldsymbol{s}\omega j|\nabla\cdot\hat{\boldsymbol{A}}|i,0\rangle\Big) e^{-i(\omega-\omega_{if})(t-t_1)} = \\
&\hbar^2 K_{\hat{\boldsymbol{A}}\cdot\hat{\boldsymbol{p}}} + 2\hbar^2\mathrm{Re}[K_{cross}] + \hbar^2 K_{\nabla\cdot\hat{\boldsymbol{A}}}\,,
\end{aligned} \tag{13}$$

where we have used the expression for the interaction Hamiltonian in Eq. 7 and further that after inserting the MQED vector potential, the modes of the electromagnetic field are labelled by ω and thus $\omega_{if\boldsymbol{M}} = \omega - \omega_{if}$, where $\hbar\omega_{if}$ is the energy difference between the initial and final state. To make the origin of the different terms clear, we have dubbed these $K_{\hat{\boldsymbol{A}}\cdot\hat{\boldsymbol{p}}}$, $\mathrm{Re}[K_{cross}]$ and $K_{\nabla\cdot\hat{\boldsymbol{A}}}$ respectively. Importantly, the two last terms only arise when the dielectric tensor of the material is anisotrpic. In an isotropic material, the condition $\nabla\cdot(\overleftrightarrow{\epsilon}\hat{\boldsymbol{A}}) = 0$ would mean that $\nabla\cdot(\hat{\boldsymbol{A}}) = 0$. We can therefore think of these extra terms as an anisotropic correction. For the TMDs considered in this work, the dielectric response is

uniaxial and these terms therefore need to be taken into account. In the following, we will derive the contribution from each of these three terms to the interaction kernel.

The $\hat{\boldsymbol{A}} \cdot \hat{\boldsymbol{p}}$ contribution

We are first going to consider the dominant $\hat{\boldsymbol{A}} \cdot \hat{\boldsymbol{p}}$ contribution to the interaction kernel,

$$K_{\hat{\boldsymbol{A}}\cdot\hat{\boldsymbol{p}}} = \frac{e^2}{m_0^2} \int d\omega \int d^3r \sum_j \left| \langle i, 0_{\boldsymbol{M}} | \hat{\boldsymbol{A}} \cdot \hat{\boldsymbol{p}} | f, \boldsymbol{r}\omega j \rangle \right|^2 e^{-i(\omega-\omega_{if})(t-t_1)} \,. \tag{14}$$

Inserting the MQED expansion of the vector potential, equation 6, into equation 14 one finds:

$$K_{\hat{\boldsymbol{A}}\cdot\hat{\boldsymbol{p}}} = \frac{4\alpha}{m_0^2 c} \int d\omega d^3r d^3s \psi_i^*(\mathbf{r}) \psi_i(\mathbf{s}) \mathrm{Im} \left[\overleftrightarrow{G}_{nm}(\mathbf{r}, \mathbf{s}, \omega) \right] \left(\hat{p}_n \cdot \psi_f(\mathbf{r}) \right) \left(\hat{p}_m \cdot \psi_f(\mathbf{s}) \right)^* e^{-i(\omega-\omega_{if})(t-t_1)} \,, \tag{15}$$

where we have used the commutation relation $\left[\hat{f}_i(\boldsymbol{r}, \omega), \hat{f}_j(\boldsymbol{r'}, \omega') \right] = \delta(\omega - \omega')\delta(\boldsymbol{r} - \boldsymbol{r'})\delta_{ij}$ [2] along with the Green's function identity:[2,3]

$$\sum_i \frac{\omega^2}{c^2} \int d^3x \, \mathrm{Im} \left[\epsilon(\mathbf{x}, \omega) \right] \left[\overleftrightarrow{G}_{ni}(\mathbf{r}, \mathbf{x}, \omega) \overleftrightarrow{G}^*_{im}(\mathbf{x}, \mathbf{s}, \omega) \right] = \mathrm{Im} \left[\overleftrightarrow{G}_{nm}(\mathbf{r}, \mathbf{s}, \omega) \right] \,. \tag{16}$$

Having arrived at Eq. 15, we next insert the explicit DGF and electronic wave functions. The subband states in the TMD are Bloch states and can be expressed as:

$$\psi(\boldsymbol{r}) = \frac{1}{\sqrt{A}} \phi_j(\boldsymbol{r}) e^{i\boldsymbol{k}\cdot\boldsymbol{\rho}} \,, \tag{17}$$

where $\boldsymbol{k}$ and $\boldsymbol{\rho}$ are the in-plane momentum and location respectively, and A is the crystal area. $\phi_j(\boldsymbol{r})$ inherits the in-plane periodicity of the lattice such that for any real space lattice vector, $\boldsymbol{R}$, $\phi_{i,f}(\boldsymbol{r} + \boldsymbol{R}) = \phi_{i,f}(\boldsymbol{r})$. This leads to the following normalization condition for the

Bloch functions,

$$1 = \int d^3r |\psi(\boldsymbol{r})|^2 = \frac{1}{A}\int d\boldsymbol{\rho} dz |\phi(\boldsymbol{r})|^2 = \frac{1}{A}\sum_{\text{u.c.}}\int_{\text{u.c.}} d\boldsymbol{\rho}\int dz|\phi(\boldsymbol{r})|^2\,, \tag{18}$$

meaning that we have to require,

$$\int_{\text{u.c.}} d\boldsymbol{\rho}\int dz|\phi(\boldsymbol{r})|^2 = A_{\text{u.c.}}\,, \tag{19}$$

where, $\sum_{\text{u.c.}}$, $\int_{\text{u.c.}}$ and $A_{\text{u.c.}}$ denote the unit cell summation, the unit cell integral and the unit cell area respectively.

Combining the DGF from the methods section of the main text with the wave functions from equation 17, we find that:

$$\begin{aligned}
&\int d\boldsymbol{r} d\boldsymbol{r'} \psi_i^*(\boldsymbol{r})\psi_i(\boldsymbol{r'})\text{Im}\left[\overleftrightarrow{G}_{kl}(\boldsymbol{r},\boldsymbol{r'};\omega)\right]\left(p_k\psi_f(\boldsymbol{r})\right)\left(p_l\psi_f(\boldsymbol{r'})\right)^* = \\
&\int d\boldsymbol{q}\frac{c^2\hbar^2}{4\pi^2\omega^2 k_z}\text{Im}\left[\frac{r_g e^{-2k_z d}-1}{r_g e^{-2k_z d}+1}\right]\times \\
&\left[\frac{k_z^2}{\epsilon_\parallel}\int d\boldsymbol{r}\int d\boldsymbol{r'}\psi_i^*(\boldsymbol{r})\psi_i(\boldsymbol{r'})e^{i\boldsymbol{q}(\boldsymbol{\rho}-\boldsymbol{\rho'})}\text{sinh}(k_z z)\text{sinh}(k_z z')\left(\partial_{\hat{q}}\psi_f(\boldsymbol{r})\right)\left(\partial_{\hat{q}}\psi_f^*(\boldsymbol{r'})\right)\right. \\
&\left.+\frac{\epsilon_\parallel q^2}{\epsilon_\perp^2}\int d\boldsymbol{r}\int d\boldsymbol{r'}\psi_i^*(\boldsymbol{r})\psi_i(\boldsymbol{r'})e^{i\boldsymbol{q}(\boldsymbol{\rho}-\boldsymbol{\rho'})}\text{cosh}(k_z z)\text{cosh}(k_z z')\left(\partial_z\psi_f(\boldsymbol{r})\right)\left(\partial_z\psi_f^*(\boldsymbol{r'})\right)\right] = \\
&\frac{1}{A^2}\int d\boldsymbol{q}\frac{c^2\hbar^2}{4\pi^2\omega^2 k_z}\text{Im}\left[\frac{r_g e^{-2k_z d}-1}{r_g e^{-2k_z d}+1}\right]\times \\
&\left[\frac{k_z^2}{\epsilon_\parallel}\left|\int d\boldsymbol{r}\phi_i(\boldsymbol{r})e^{i(\boldsymbol{q}-\boldsymbol{k}_i+\boldsymbol{k}_f)\cdot\boldsymbol{\rho}}\text{sinh}(k_z z)\left(\partial_{\hat{q}}\phi_f(\boldsymbol{r})+(\boldsymbol{k_f}\cdot\hat{\boldsymbol{q}})\phi_f(\boldsymbol{r})\right)\right|^2\right. \\
&\left.+\frac{\epsilon_\parallel q^2}{\epsilon_\perp^2}\left|\int d\boldsymbol{r}\phi_i(\boldsymbol{r})e^{i(\boldsymbol{q}-\boldsymbol{k_i}+\boldsymbol{k}_f)\cdot\boldsymbol{\rho}}\text{cosh}(k_z z)\left(\partial_z\phi_f(\boldsymbol{r})\right)\right|^2\right]\,.
\end{aligned} \tag{20}$$

Assuming that the phase factor, $e^{-i(\boldsymbol{q}-\boldsymbol{k}_f-\boldsymbol{k}_f)\cdot\boldsymbol{\rho}}$, varies slowly inside of a single unit cell leads

to conservation of in-plane momentum:

$$\int d\boldsymbol{\rho} e^{i(\boldsymbol{q}-\boldsymbol{k}_i+\boldsymbol{k}_f)\cdot\boldsymbol{\rho}}\phi_i(\boldsymbol{r})\phi_f^*(\boldsymbol{r}) \approx A_{u.c}\sum_{\mathrm{n}=1}^{N_{\mathrm{u.c.}}} e^{i(\boldsymbol{q}-\boldsymbol{k}_i+\boldsymbol{k}_f)\cdot\boldsymbol{\rho}_n}\frac{1}{A_{u.c}}\int_{\mathrm{u.c.}} d\boldsymbol{\rho}\phi_i(\boldsymbol{r})\phi_f^*(\boldsymbol{r})$$
$$= \frac{A}{A_{\mathrm{u.c}}}\delta_{\boldsymbol{k}_f,\boldsymbol{k}_f+\boldsymbol{q}}\int_{\mathrm{u.c.}} d\boldsymbol{\rho}\phi_i(\boldsymbol{r})\phi_f^*(\boldsymbol{r})\,. \tag{21}$$

This in turn means that equation 20 can be recast as:

$$\int d\boldsymbol{r}d\boldsymbol{r'}\psi_i^*(\boldsymbol{r})\psi_i(\boldsymbol{r'})\mathrm{Im}\left[\overleftrightarrow{G}_{kl}(\boldsymbol{r},\boldsymbol{r'};\omega)\right](p_k\psi_f(\boldsymbol{r}))(p_l\psi_f(\boldsymbol{r'}))^* =$$
$$\int d\boldsymbol{q}\frac{c^2\hbar^2}{4\pi^2\omega^2k_zA_{\mathrm{u.c.}}^2}\delta_{\boldsymbol{k}_f,\boldsymbol{k}_i+\boldsymbol{q}}\mathrm{Im}\left[\frac{r_ge^{-2k_zd}-1}{r_ge^{-2k_zd}+1}\right]\times$$
$$\left[\frac{\epsilon_\parallel q^2}{\epsilon_\perp^2}\left|\int_{\mathrm{u.c.}} d\boldsymbol{\rho}\int dz\phi_i(\boldsymbol{r})\cosh(k_zz)\left(\partial_z\phi_f(\boldsymbol{r})\right)\right|^2\right.$$
$$\left.+\frac{k_z^2}{\epsilon_\parallel}\left|\int d\boldsymbol{r}\phi_i(\boldsymbol{r})\sinh(k_zz)\left(\partial_{\hat{q}}\phi_f(\boldsymbol{r})+(\boldsymbol{k_f}\cdot\hat{\boldsymbol{q}})\phi_f(\boldsymbol{r})\right)\right|^2\right]\,. \tag{22}$$

Inserting equation 22 into equation 15 therefore completes the calculation of $K_{\hat{\boldsymbol{A}}\cdot\hat{\boldsymbol{p}}}$. Looking at equation 10, we see that when inserting the matrix elements into the equation of motion there is an additional sum over the final states, f. Because of the delta function in equation 22, the possible final states are restricted to the states along the dispersion relation obeying $\boldsymbol{q}=\boldsymbol{k}_f-\boldsymbol{k}_i$ and we can thus also label the transition frequency between the initial and final state by the $\boldsymbol{q}$ such that, $\omega_{if}=\omega_{if}(\boldsymbol{q})\equiv\omega_{if\boldsymbol{q}}$. This explains the form of the phase factor in equation 11. With this consideration, the $\hat{\boldsymbol{A}}\cdot\hat{\boldsymbol{p}}$ contribution to the interaction kernel in equation 11 can be written as,

$$K_{\hat{\boldsymbol{A}}\cdot\hat{\boldsymbol{p}}}(\boldsymbol{q},\omega) = \frac{\alpha c\hbar^2}{m^2\pi^2\omega^2k_zA_{\mathrm{u.c.}}^2}\mathrm{Im}\left[\frac{r_ge^{-2k_zd}-1}{r_ge^{-2k_zd}+1}\right]$$
$$\left[\frac{\epsilon_\parallel q^2}{\epsilon_\perp^2}\left|\int_{\mathrm{u.c.}} d\boldsymbol{\rho}\int dz\phi_i(\boldsymbol{r})\cosh(k_zz)\left(\partial_z\phi_f(\boldsymbol{r})\right)\right|^2\right.$$
$$\left.+\frac{k_z^2}{\epsilon_\parallel}\left|\int d\boldsymbol{r}\phi_i(\boldsymbol{r})\sinh(k_zz)\left(\partial_{\hat{q}}\phi_f(\boldsymbol{r})+(\boldsymbol{k_f}\cdot\hat{\boldsymbol{q}})\phi_f(\boldsymbol{r})\right)\right|^2\right]\,. \tag{23}$$

Anisotropic corrections

We next to turn to the anisotropic correction terms. In order to analyze these terms, we need the derivative of the MQED vector potential in Eq. 6,

$$\nabla \cdot \hat{\boldsymbol{A}}(\mathbf{r}) = \sqrt{\frac{\hbar}{\pi\epsilon_0}} \int d\omega \frac{\omega}{c^2} \int d^3 s\, \partial_i \overleftrightarrow{G}_{ij}(\mathbf{r}, \mathbf{s}, \omega) \sqrt{\mathrm{Im}\, \epsilon_{jj}(\mathbf{s}, \omega)} \hat{f}_j(\mathbf{s}, \omega) + \mathrm{h.c.}\,, \tag{24}$$

where we have used Einstein summation convention in the above. We note again here that in an isotropic media, it can be shown for the DGF discussed in the main text that $\partial_i \overleftrightarrow{G}_{ij}(\mathbf{r}, \mathbf{s}, \omega) = 0$ for every excitation orientation j. In that case only the $K_{\hat{\boldsymbol{A}}\cdot\hat{\boldsymbol{p}}}$ term would thus survive. However, because of the uniaxial nature of the TMD's dielectric tensor we need to explicitly consider the extra terms.

By substituting the equation above into the expression of $K_{\nabla\hat{\boldsymbol{A}}}$, we find

$$K_{\nabla\cdot\hat{\boldsymbol{A}}} = \frac{e^2}{4m^2} \frac{\hbar}{\pi\epsilon_0} \sum_f \int d\omega \frac{\omega^2}{c^4} \int d\boldsymbol{s} \sum_j \mathrm{Im}\,(\epsilon_{jj}) \int d\boldsymbol{r} \psi_i^*(\boldsymbol{r}) \psi_f(\boldsymbol{r}) \partial_i \overleftrightarrow{G}_{ij}(\mathbf{r}, \mathbf{s}, \omega) \times$$
$$\int d\boldsymbol{r'} \psi_i(\boldsymbol{r'}) \psi_f^*(\boldsymbol{r'}) (\partial'_k \overleftrightarrow{G}_{kj}(\mathbf{r}', \mathbf{s}, \omega))^* \, e^{-i(\omega-\omega_{if})(t-t_1)}\,. \tag{25}$$

where the symbol ∂' corresponds to the partial derivative with respect to the $\boldsymbol{r'}$ coordinates. In order to continue we shall use two Green's function identities. First, we use $(\overleftrightarrow{G}_{kj}(\mathbf{r}', \mathbf{s}, \omega))^* = \overleftrightarrow{G}_{jk}(\mathbf{s}, \mathbf{r}', \omega)$ to show that $(\partial'_k \overleftrightarrow{G}_{kj}(\mathbf{r}', \mathbf{s}, \omega))^* = \partial'_k (\overleftrightarrow{G}_{kj}(\mathbf{r}', \mathbf{s}, \omega))^* = \partial'_k \overleftrightarrow{G}_{jk}(\mathbf{s}, \mathbf{r}', \omega)$, since the derivative is on a real parameter. Second, we use the identity in Eq. (15), to show that

$$\sum_j \frac{\omega^2}{c^2} \int d\boldsymbol{s}\, \mathrm{Im}\,(\epsilon_{jj}) \partial_i \overleftrightarrow{G}_{ij}(\mathbf{r}, \mathbf{s}, \omega) (\partial'_k \overleftrightarrow{G}_{kj}(\mathbf{r}', \mathbf{s}, \omega))^* = \partial_i \partial'_k \mathrm{Im} \left[\overleftrightarrow{G}_{ik}(\mathbf{r}, \mathbf{r}', \omega) \right]. \tag{26}$$

Therefore, we find the compact formula,

$$K_{\nabla\cdot\hat{\boldsymbol{A}}} = \frac{\alpha\hbar^2}{m^2 c}\sum_f \int d\omega \int d\boldsymbol{r}\psi_i^*(\boldsymbol{r})\psi_f(\boldsymbol{r}) \int d\boldsymbol{r'}\psi_i(\boldsymbol{r'})\psi_f^*(\boldsymbol{r'})\partial_i\partial_k'\mathrm{Im}\left[\overleftrightarrow{G}_{ik}(\mathbf{r},\mathbf{r}',\omega)\right] e^{-i(\omega-\omega_{if})(t-t_1)}\,, \tag{27}$$

The next step is to insert the specific expression for the DGF and the wave functions. This procedure is very similar to the procedure for the $\hat{\boldsymbol{A}}\cdot\hat{\boldsymbol{p}}$ term and in the end one ends up with the following result:

$$\begin{aligned}
&K_{\nabla\cdot\hat{\boldsymbol{A}}} = \frac{\alpha\hbar^2}{m^2 c}\sum_f \int d\omega \int d\boldsymbol{r}\psi_i^*(\boldsymbol{r})\psi_f(\boldsymbol{r}) \int d\boldsymbol{r'}\psi_i(\boldsymbol{r'})\psi_f^*(\boldsymbol{r'})\times \\
&\frac{c^2(\epsilon_\parallel-\epsilon_\perp)^2}{4\pi^2\omega^2\epsilon_\parallel\epsilon_\perp^2}\int d\boldsymbol{q}k_z^2q^2\mathrm{Im}\left[\frac{r_g e^{-2k_z d}-1}{r_g e^{-2k_z d}+1}\right]\sinh(k_z z)\sinh(k_z z')e^{-i\omega_{if\boldsymbol{M}}(t-t_1)} = \\
&\frac{\alpha\hbar^2 c(\epsilon_\parallel-\epsilon_\perp)^2}{4\pi^2 m^2\epsilon_\parallel\epsilon_\perp^2}\int d\omega\int d\boldsymbol{q}\,\delta_{\boldsymbol{k}_f,\boldsymbol{k}_i+\boldsymbol{q}}\frac{k_z^2q^2}{\omega^2}\mathrm{Im}\left[\frac{r_g e^{-2k_z d}-1}{r_g e^{-2k_z d}+1}\right]\times \\
&\left|\frac{1}{A_{\text{u.c.}}}\int_{\text{u.c.}} d\boldsymbol{r}\phi_i^*(\boldsymbol{r})\phi_f(\boldsymbol{r})\sinh(k_z z)\right|^2 e^{-i(\omega-\omega_{if})(t-t_1)}\,.
\end{aligned} \tag{28}$$

By the same argument as for the $\hat{\boldsymbol{A}}\cdot\hat{\boldsymbol{p}}$ term above, we can write the contribution to the integral kernel, $K(\boldsymbol{q},\omega)$, in equation 11 as:

$$\begin{aligned}
&K_{\nabla\cdot\hat{\boldsymbol{A}}}(\boldsymbol{q},\omega) = \\
&\frac{\alpha\hbar^2 c(\epsilon_\parallel-\epsilon_\perp)^2}{4\pi^2 m^2\epsilon_\parallel\epsilon_\perp^2}\frac{k_z^2q^2}{\omega^2}\mathrm{Im}\left[\frac{r_g e^{-2k_z d}-1}{r_g e^{-2k_z d}+1}\right]\times\left|\frac{1}{A_{\text{u.c.}}}\int_{\text{u.c.}} d\boldsymbol{r}\phi_i^*(\boldsymbol{r})\phi_f(\boldsymbol{r})\sinh(k_z z)\right|^2
\end{aligned} \tag{29}$$

As expected, the kernel contribution in equation 29 is equal to zero in an isotropic medium where $\epsilon_\perp = \epsilon_\parallel$. We further note that the contribution is generally positive.

In order to find the final term of the kernel, K_{cross}, we can use a similar procedure as

was done above. Consequently,

$$K_{cross} =$$
$$\frac{2\alpha\hbar^2}{m^2c}\sum_f \int d\omega \int d\boldsymbol{r}\psi_i^*(\boldsymbol{r})\psi_f(\boldsymbol{r}) \int d\boldsymbol{r'}\partial_k'\psi_i(\boldsymbol{r'})\psi_f^*(\boldsymbol{r'})\partial_i \mathrm{Im}\left[\overleftrightarrow{G}_{ik}(\mathbf{r},\mathbf{r}',\omega)\right] e^{-i(\omega-\omega_{if})(t-t_1)} . \tag{30}$$

When substituting in the Green's function and the wave functions, we now find:

$$K_{cross} = \frac{\alpha\hbar^2 c}{2\pi^2 m^2\epsilon_\perp}\int d\omega \frac{1}{\omega^2}\int d\boldsymbol{q}\mathrm{Im}\left[\frac{r_g e^{-2k_z d}-1}{r_g e^{-2k_z d}+1}\right]\delta_{\boldsymbol{k}_f,\boldsymbol{k}_i+\boldsymbol{q}}\left(\frac{1}{A_{\mathrm{u.c.}}}\int_{\mathrm{u.c.}} d\boldsymbol{r}\phi_i^*(\boldsymbol{r})\phi_f(\boldsymbol{r})\sinh(k_z z)\right)\times$$
$$\left(-iqk_z(\frac{\epsilon_\perp}{\epsilon_\parallel}-1)\frac{1}{A_{\mathrm{u.c.}}}\int_{\mathrm{u.c.}} d\boldsymbol{r}\partial_{\hat{\boldsymbol{q}}}\phi_i(\boldsymbol{r})\phi_f^*(\boldsymbol{r})\sinh(k_z z)\right.$$
$$\left.+q^2(\frac{\epsilon_\parallel}{\epsilon_\perp}-1)\frac{1}{A_{\mathrm{u.c.}}}\int_{\mathrm{u.c.}} d\boldsymbol{r}\partial_z\phi_i(\boldsymbol{r})\phi_f^*(\boldsymbol{r})\cosh(k_z z)\right) e^{-i(\omega-\omega_{if})(t-t_1)} . \tag{31}$$

Finally, this means that we can write the final contribution to the integral kernel as,

$$K_{cross}(\boldsymbol{q},\omega) = \frac{\alpha\hbar^2 c}{2\pi^2 m^2\epsilon_\perp}\frac{1}{\omega^2}\mathrm{Im}\left[\frac{r_g e^{-2k_z d}-1}{r_g e^{-2k_z d}+1}\right]\left(\frac{1}{A_{\mathrm{u.c.}}}\int_{\mathrm{u.c.}} d\boldsymbol{r}\phi_i^*(\boldsymbol{r})\phi_f(\boldsymbol{r})\sinh(k_z z)\right)\times$$
$$\left(-iqk_z(\frac{\epsilon_\perp}{\epsilon_\parallel}-1)\frac{1}{A_{\mathrm{u.c.}}}\int_{\mathrm{u.c.}} d\boldsymbol{r}\partial_{\hat{\boldsymbol{q}}}\phi_i(\boldsymbol{r})\phi_f^*(\boldsymbol{r})\sinh(k_z z)\right.$$
$$\left.+q^2(\frac{\epsilon_\parallel}{\epsilon_\perp}-1)\frac{1}{A_{\mathrm{u.c.}}}\int_{\mathrm{u.c.}} d\boldsymbol{r}\partial_z\phi_i(\boldsymbol{r})\phi_f^*(\boldsymbol{r})\cosh(k_z z)\right) . \tag{32}$$

Again, as expected, the kernel contribution in equation 32 is equal to zero in an isotropic medium where $\epsilon_\perp = \epsilon_\parallel$. We further note that this term can be negative, though the overall sum $K_{\hat{\boldsymbol{A}}\cdot\hat{\boldsymbol{p}}} + 2\mathrm{Re}[K_{cross}] + K_{\nabla\cdot\hat{\boldsymbol{A}}}$ must be larger than 0.

Common approximations

The kernel function, $K(\boldsymbol{q},\omega)$ and equation 10 fully describe the interaction of the system with its environment and the corresponding dynamics. The work is therefore in principle done at this point. However, we now take few limits to compare with commonly used approximations.

Assuming that the emission takes place in the weak coupling regime, it is fair to assume that $C_i(t')$ is slowly varying relative to the phase factor. We can therefore replace $C_i(t')$ with $C_i(t)$ in equation 11 and extend the upper limit of the time integral to infinity (the Markov approximation). This leads to the following 1st order approximate expression for the rate:

$$\Gamma_{\text{1st order MQED}} = 2\pi \int d\boldsymbol{q} K(\omega_{if\boldsymbol{q}}, \boldsymbol{q}) \,. \tag{33}$$

To investigate the effect of the nonlocality of the matrix elements and transition energy, we can neglect the dispersion of the subband states. To this end, we can perform calculations with a $\boldsymbol{q}$ independent transition energy $\omega_{if\boldsymbol{q}} = \omega_{if\boldsymbol{q}=0}$ and neglect the nonlocality of matrix elements. While this approximation neglects the dispersion of the electronic states it still treats the light-matter interaction beyond the dipole approximation. The results within this approximation are the green lines in Fig. 5 of the main text.

A different set of approximations can come when considering the dynamics within a local point dipole approximation. This approximation requires the assumption that the spatial variation of the field is negligible across the TMD slab. In such approximation, the $\cosh(k_z z)$ and $\sinh(k_z z)$ factors can therefore be pulled outside of the integrals in equation 23, and replaced with their values in the middle of the slab. This means that the integrals whose integrand does not include a derivative of the wave functions all vanish due to the orthogonality of the subband state wave functions. This in effect removes both the $\mathrm{Re}[K_{cross}]$ and the $K_{\nabla\cdot\hat{\boldsymbol{A}}}$ terms. Further, in this local approximation, we do not consider the dispersion of the transition energy or the nonlocality of the matrix elements, that is $\omega_{if\boldsymbol{q}} = \omega_{if\boldsymbol{q}=0}$. As

a result, the coupling kernel becomes:

$$K(\boldsymbol{q},\omega) = \frac{\alpha c\hbar^2}{m^2\pi^2\omega^2 k_z A_{\text{u.c.}}^2}\text{Im}\left[\frac{r_g e^{-2k_z d}-1}{r_g e^{-2k_z d}+1}\right]$$
$$\left[\frac{\epsilon_\parallel q^2\cosh^2(k_z d/2)}{\epsilon_\perp^2}\left|\langle f_{\boldsymbol{q}=0}|\hat{p}_z|i\rangle\right|^2 + \frac{k_z^2\sinh^2(k_z d/2)}{\epsilon_\parallel}\left|\langle f_{\boldsymbol{q}=0}|\hat{p}_x|i\rangle\right|^2\right] , \tag{34}$$

where $|f_{\boldsymbol{q}=0}\rangle$ denotes the final state that would result from a vertical transition between the subbands.

The final approximation is what we denote as the sheet dipole approximation. In this approximation we again assume that the $\cosh(k_z z)$ and $\sinh(k_z z)$ factors can be replaced by their values in the middle of the slab. However, we now retain the dispersion of the transition energy and the nonlocality of the matrix elements. The sheet dipole approximation is therefore equivalent to calculations with the following kernel:

$$K(\boldsymbol{q},\omega) = \frac{\alpha c\hbar^2}{m^2\pi^2\omega^2 k_z A_{\text{u.c.}}^2}\text{Im}\left[\frac{r_g e^{-2k_z d}-1}{r_g e^{-2k_z d}+1}\right]$$
$$\left[\frac{\epsilon_\parallel q^2\cosh^2(k_z d/2)}{\epsilon_\perp^2}\left|\langle f_{\boldsymbol{q}}|\hat{p}_z|i\rangle\right|^2 + \frac{k_z^2\sinh^2(k_z d/2)}{\epsilon_\parallel}\left|\langle f_{\boldsymbol{q}}|\hat{p}_x|i\rangle\right|^2\right] . \tag{35}$$

Supplementary note 2: Dipole moment scaling of rates

When the dominant contributions to the decay rate come from excitations whose q values fulfill the condition $q \ll 1/d$ (d is the QW width), the coupling should be well described within the dipole approximation. Within the dipole approximation, the decay rates only depend on the wave functions of the subbands via their effective transition dipole moment. Specifically, the rate depends proportionally on the absolute square of the effective dipole moments, $|\boldsymbol{\mu}_{if}|^2$. In general, the transition dipole moment is a nonlocal quantity and thus it has a dependence on the value of q. However, if all of the relevant excitations live at relatively small q-values, it should provide a decent approximation to say that the decay rates are directly proportional to the absolute square of the transition dipole moments at $q = 0$,

$$\Gamma_{\text{Point dipole}} \propto |\mu_{if\mathbf{q}=0}|^2 \,. \tag{36}$$

That means that if the we rescale the rates from the 1D wave function approximations by the ratio between the absolute square of their transition dipole moment and the absolute square of the transition dipole moment from the full 3D wave function approximations, the rates should agree, as long as $q \ll 1/d$ and thus $|\mu_{if\mathbf{q}}|^2 \approx |\mu_{if\mathbf{q}=0}|^2$. In the following we thus investigate how rescaling the rates calculated within the 1D wave function approximations according to,

$$\Gamma^{\text{1D}}_{\text{scaled}} = \frac{|\mu^{\text{3D}}_{if\boldsymbol{q}=0}|^2}{|\mu^{\text{1D}}_{if\boldsymbol{q}=0}|^2}\Gamma^{\text{1D}} \,, \tag{37}$$

works towards accounting for the differences between the different wave function approximations when all relevant excitations obey $q \ll 1/d$. In the above, $\mu^{\text{3D(1D)}}_{if\boldsymbol{q}}$ is the transition dipole moment at $q = 0$ when using the 3D(1D) wave functions, Γ^{1D} is the decay rate obtained with the particular 1D wave function approximation and $\Gamma^{\text{1D}}_{\text{scaled}}$ is the scaled version

of the rate.

Figure 1 a(b) shows the decay rates calculated within the three different wave function approximations alongside the rescaled versions for 2(3) layers of WSe_2. For both the 2 and 3 layer slab the condition that $q \ll 1/d$ for all of the relevant excitations holds relatively well, since the coupling to the plasmon makes up the dominant contribution to the decay rates within the range of graphene Fermi levels considered in this work. We observe that for both the 2 and 3 layer slabs, the rescaling of the rates by the ratio of the transition dipole moments leads to good agreement between the different approximations. The difference between the 1D DFT and 3D DFT is almost entirely explained by the difference in their effective dipole moments. For the Particle in a box wave functions the picture is slightly more complicated.

Starting with the 3 layer slab in figure 1b, we observe that generally the rescaling works well, with a tendency to worsen as the graphene fermi level is increased. The deviations that start to appear at larger graphene Fermi levels stem from the fact that we increasingly observe more coupling to the electron-hole continuum of graphene which doesn't obey $q \ll 1/d$. The extreme example of this are the huge qualitative differences in the rates that we observe for the 5 layer slab in figure 6(b) of the main text, where the coupling to the electron-hole continuum is dominant at larger graphene Fermi levels. For the 2 layer slab, we observe that the rescaling of the Particle in a box states slightly overshoots the rates calculated using the 3D DFT wave functions, but that the qualitative trends agree well. The reason for the overshoot is down to nonlocality of the transition dipole moment near where the plasmon intersects the transition energy dispersion. This nonlocality of the dipole moment near the plasmon is far more pronounced for the 2 layer slabs than for the thicker samples, because, as can be seen from figure 3 in the main text, the larger transition energies in the 2 layer slabs means that the plasmon intersects the transition energy dispersion at far larger q than what is the case for the thicker samples. This nonlocality doesn't manifest as

much in the difference between the 1D and 3D DFT wave functions, because the difference in their effective dipoles in the out-of-plane direction is a constant factor independent of q. This happens because the 1D DFT wave functions retain the out-of-plane character of the 3D DFT states, and therefore they differ from the full 3D states only in that they miss the in-plane overlap of the states.

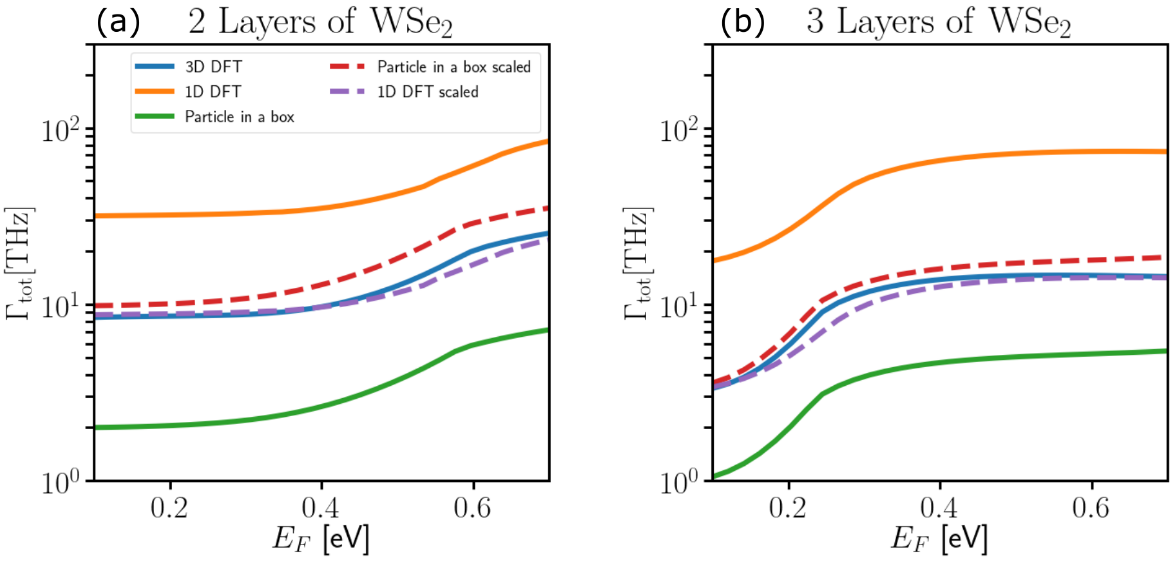

Supplementary Figure 1: The scaling of the dipole approximated result to fit the full 3D DFT wavefunction transition. This figure shows the scaling of the first order decay rates by the square of the ratio between the vertical dipole transitions matrix elements, $|\langle f_{\mathbf{q}=0}|\hat{p}_z|i\rangle|^2$, between the different wave function approximations.

Supplementary note 3: Reflection from a uniaxial material

In this section we will derive the Fresnel reflection coefficient off of the graphene sheet for a wave incident on the graphene sheet from a uniaxial medium. The incident wave with magnitude, E_0, is defined as,

$$\boldsymbol{E_i} = E_0 \left(\frac{\epsilon_\perp k_z}{k_N} \hat{x} - \frac{\epsilon_\| q}{k_N} \hat{z} \right) e^{ik_z z + iqx} \,, \tag{38}$$

where q is the wave vector in the x-direction and $k_z = \sqrt{\epsilon_x k_0^2 - \frac{\epsilon_x}{\epsilon_z} q^2}$ is the out-of-plane wave number. With these definitions, the incident wave satisfies $\boldsymbol{\nabla} \cdot \left[\underline{\underline{\epsilon}} \cdot \boldsymbol{E} \right] = 0$ and $k_0^2 = \frac{\omega^2}{c^2} = \frac{q^2}{\epsilon_\perp} + \frac{k_z^2}{\epsilon_\|}$ by defining $k_N^2 = (\epsilon_\perp k_z)^2 + (\epsilon_\| q)^2$.

Defining the reflection-, r_g, and transmission coefficient, t_g, the expressions for the reflected and transmitted fields become:

$$\boldsymbol{E}_r = r_g E_0 \left(\frac{\epsilon_\perp k_z}{k_N} \hat{x} + \frac{\epsilon_\| q}{k_N} \hat{z} \right) e^{-ik_z z + iqx} \,, \tag{39}$$

$$\boldsymbol{E}_t = t_g E_0 \left(\frac{k_{z,t}}{k_0} \hat{x} - \frac{q}{k_0} \hat{z} \right) e^{ik_{z,t} z + iqx} \,, \tag{40}$$

where the z-component of the wave vector for the transmitted field is $k_{z,t} = (k_0^2 - q^2)^{1/2}$.

Restricting the discussion to non-magnetic materials, $\mu_r = 1$, the magnetic field, $\boldsymbol{H}$, and the magnetic flux density, $\boldsymbol{B}$ are trivially connected via $\boldsymbol{H} = \frac{1}{\mu_0} \boldsymbol{B}$. This means that the magnetic field can be calculated directly from the electric field via Faraday's law,

$\boldsymbol{H} = \frac{1}{i\mu_0\omega}\boldsymbol{\nabla} \times \boldsymbol{E}$:

$$\boldsymbol{H}_i = \hat{y}\frac{\epsilon_\perp \epsilon_\| k_0^2 E_0}{k_N \omega \mu_0} e^{ik_z z + iqx}\,, \tag{41}$$

$$\boldsymbol{H}_r = -\hat{y} r \frac{\epsilon_\perp \epsilon_\| k_0^2 E_0}{k_N \omega \mu_0} e^{iqx - ik_z z}\,, \tag{42}$$

$$\boldsymbol{H}_t = \hat{y} t \frac{E_0 k_0}{\omega \mu_0} e^{i(qx + k_{z,t} z)}\,. \tag{43}$$

The next step is to use the electromagnetic boundary conditions. Specifically, the boundary conditions on the parallel parts of the fields. Noting that the unit normal of the interface is $\hat{z}$ and that the surface current can be related to the transmitted field via Ohm's law,

$$\boldsymbol{K} = \sigma(\boldsymbol{q}, \omega) E_{t,x}(z = 0)\hat{x}\,, \tag{44}$$

where $\sigma(\boldsymbol{q}, \omega)$ is the nonlocal conductivity of graphene as described in Ref.,[4] the boundary conditions at the interface becomes:

$$E_{t,x}(z = 0) = E_{i,x}(z = 0) + E_{r,x}(z = 0) \tag{45}$$

$$\hat{z} \times (\boldsymbol{H}_t(z = 0) - \boldsymbol{H}_i(z = 0) - \boldsymbol{H}_r(z = 0)) = \sigma(\boldsymbol{q}, \omega) E_{t,x}(z = 0)\hat{x}\,. \tag{46}$$

Inserting the electric fields into equation 45 leads to:

$$t_g = (1 + r_g)\frac{\epsilon_\perp k_z k_0}{k_N k_{z,t}}\,, \tag{47}$$

and inserting the magnetic fields into equation 46 gives:

$$(1 - r_g)\frac{\epsilon_\perp \epsilon_\| k_0^2}{k_N} = t_g \left(\frac{k_{z,t} \omega \mu_0 \sigma(q, \omega)}{k_0} + k_0 \right)\,. \tag{48}$$

Equations 47 and 48 represent two linear equations with two unknowns and they can thus

be solved for the generalized reflection coefficient of the interface, r_g. If we consider the quasi-static limit, which is well justified here since all of the relevant excitations are strongly confined, we can take $k_z \approx i\sqrt{\frac{\epsilon_{\parallel}}{\epsilon_{\perp}}}q$. Using this, one finds the following expression for the Fresnel reflection coefficient for a wave incident on a graphene sheet from a uniaxial medium:

$$r_g = \frac{(\epsilon_{\perp}\epsilon_{\parallel})^{1/2} - 1 - i\frac{q\sigma(q,\omega)}{\epsilon_0\omega}}{(\epsilon_{\perp}\epsilon_{\parallel})^{1/2} + 1 + i\frac{q\sigma(q,\omega)}{\epsilon_0\omega}}\,. \tag{49}$$

Supplementary note 4: Calculating the diamagnetic $\hat{\boldsymbol{A}}^2$ term.

In this work, we neglect the A^2 term in the minimal coupling Hamiltonian. To provide at quantitative justification for this assumption, we here calculate it's value for the graphene-TMD-mirror cavity system and compare it to the other characteristic energies in the system.

Including the A^2 term would lead to the following addition to the Hamiltonian:

$$H_{A^2} = \frac{e^2}{2m}\hat{\boldsymbol{A}}(\boldsymbol{r})^2\,. \tag{50}$$

To calculate the above term we need to use the MQED expansion of the vector potential from equation 2 in the main text. For the configuration considered in this work, the relevant terms in the above contribution comes when a field creation and annihilation operator are combined. In such cases, the terms lead to an interaction of the electronic state with itself via the electromagnetic field causing an energy shift of the electronic states. For the excited state this energy shift would take the form:

$$\Delta E_e = \langle e,0|\frac{e^2}{2m}\hat{\boldsymbol{A}}(\boldsymbol{r})^2|e,0\rangle\,. \tag{51}$$

Substituting the expression for the MQED vector potential into equation 51 one finds,

$$\langle e,0|\frac{e^2}{2m}\hat{\boldsymbol{A}}(\boldsymbol{r})^2|e,0\rangle = \frac{1}{2m}\frac{\hbar}{\pi\epsilon_0 c^2}\langle e|\int d\omega \mathrm{Tr}\left[\mathrm{Im}\,\overleftrightarrow{\boldsymbol{G}}(\boldsymbol{r},\boldsymbol{r},\omega)\right]|e\rangle\,, \tag{52}$$

where $\mathrm{Tr}\left[\mathrm{Im}\,\overleftrightarrow{\boldsymbol{G}}\right]$ denotes the spatial trace of the DGF. Using equation 18 from the main text, one finds that:

$$\mathrm{Tr}\left[\mathrm{Im}\,\overleftrightarrow{\boldsymbol{G}}(\boldsymbol{r},\boldsymbol{r},\omega)\right] = \frac{c^2}{4\pi^2\omega^2\epsilon_\parallel}\int d\boldsymbol{q}\mathrm{Im}\left[\frac{r_g e^{-2k_z d}-1}{r_g e^{-2k_z d}+1}\right]\left(k_z\sinh^2(k_z z)+\left(\frac{\epsilon_\parallel}{\epsilon_\perp}\right)^2\frac{q^2}{k_z}\cosh^2(k_z z)\right)\,. \tag{53}$$

Inserting equation 53 into 52, while assuming azimuthal symmetry of the dispersion of the transition energy, leads to the final expression for the correction from the A^2 term:

$$\langle e,0|\frac{e^2}{2m}\hat{\boldsymbol{A}}(\boldsymbol{r})^2|e,0\rangle = \frac{\alpha\hbar^2 c}{\pi\epsilon_\parallel m A_{u.c}}\int d\omega\int dq\frac{q}{\omega^2}\mathrm{Im}\left[\frac{r_g e^{-2k_z d}-1}{r_g e^{-2k_z d}+1}\right]\int d^3r|\psi_e(\boldsymbol{r})|^2\left(k_z\sinh^2(k_z z)+\left(\frac{\epsilon_\parallel}{\epsilon_\perp}\right)^2\frac{q^2}{k_z}\cosh^2(k_z z)\right) \tag{54}$$

For the systems considered here, the value of the A^2 term depends on the thickness of the slabs. For 5 layer and 2 layer slabs the values are 0.2 meV and 1 meV respectively.

Supplementary note 5: Lamb shift renormalization of the intersubband transition energy

In this section we outline how the Lamb shift, $\delta\omega$, of the transition energies caused by the light-matter interaction can be calculated within the presented formalism. The discussion

includes the main steps from the derivation in section supplementary note 2 in the supplementary information of.[1]

The fundamental equation that we start with is the integro-differential Eq. (5) in the main text that describes the full time dynamics of the excited state. We then describe this equation with new initial state amplitudes $\tilde{C}_i(t) = C_i(t)e^{i\delta\omega t}$, to find

$$\dot{\tilde{C}}_i(t) = i\delta\omega\tilde{C}_i(t) + \int_0^t dt' \int d\omega \int d\boldsymbol{q} K(\omega, \boldsymbol{q}) e^{-i(\omega-\omega_{if}(\boldsymbol{q})-\delta\omega)(t-t')} C_i(t'). \tag{55}$$

From here, the Markov approximation is used, which assumes that $\tilde{C}_i$ is slowly varying in time compared to the rest of the time integrand, $\int d\omega \int d\boldsymbol{q} K(\omega, \boldsymbol{q}) e^{-i(\omega-\omega_{if}(\boldsymbol{q})-\delta\omega)t}$. As a result, the time integral in the equation above can be taken to infinity. When comparing the real and imaginary parts of the resulted equation, we find the expression to the spontaneous emission rate which is equation 33 (Eq. (8) in the main text), and the energy renormalization as:

$$\delta\omega = \mathcal{P}\left\{\int d\omega \int d\mathbf{q}\, \frac{K(\omega, \boldsymbol{q})}{\omega - \omega_{if}(\boldsymbol{q}) - \delta\omega}\right\} \tag{56}$$

As representative examples, figures 2 and 3 show the Lamb shifts as a function of graphene Fermi level for the 2 and 5 layer slabs of WSe_2. We see that the maximal Lamb shifts in the two cases represent less than 0.4 % of the bare transition energies of 651 meV and 84 meV respectively. We thus deduce that the dressing of the subband states by the light-matter interaction is minimal for the systems considered here.

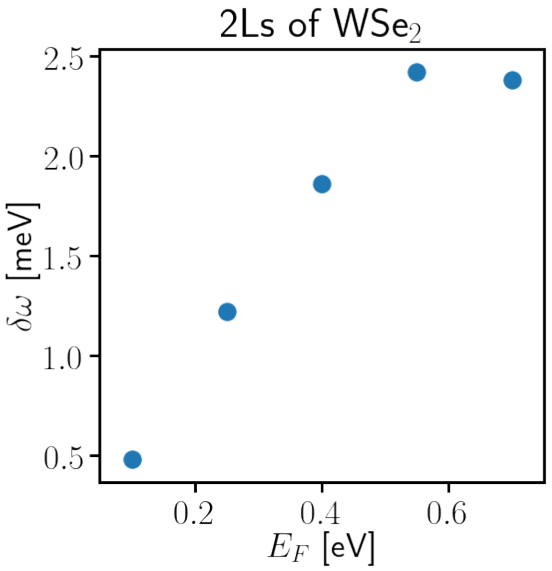

Supplementary Figure 2: Lamb shift of the intersubband transition energy as a function of the graphene Fermi level for a 2 layer slab of WSe_2.

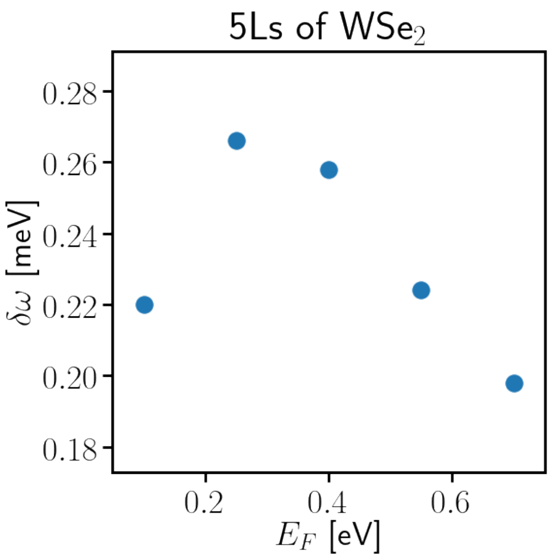

Supplementary Figure 3: Lamb shift of the intersubband transition energy as a function of the graphene Fermi level for a 5 layer slab of WSe_2.

Supplementary note 6: Examples of time dependent excited state probabilities

In order to access the time dynamics of the excited state probability we have to numerically integrate equation (5) from the main text. However, equation (5) is a stiff differential equation, as it contains dynamic on two different time scales defined by the Rabi-oscillations and the overall decay. In order to ensure numerical stability of the numerical integration scheme it is therefore necessary to use a relatively small time spacing. In order to ensure stability, we employ a time spacing of $dt = 10^{-5}$ps which is orders of magnitude smaller than the fastest time scale of the dynamics. Furthermore, we propagate everything 10^6 steps out to 10ps, which is long enough to ensure that all dynamics is well decayed for all configurations considered in this work.

In figures 4 and 5 we show the full time dependence of the excited state probabilities for a 2 and 5 layer WSe_2 slab at the graphene Fermi level resulting in the largest Purcell en-

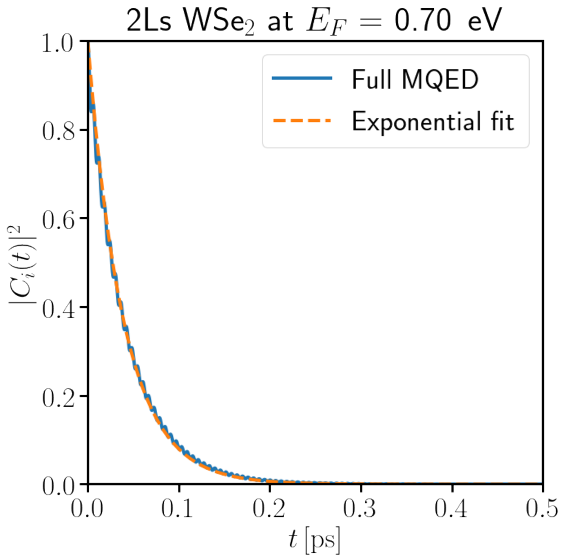

Supplementary Figure 4: Example of the time dynamics of the excited state probability for a 2 layer slab of WSe_2 at a graphene Fermi level of 0.7 eV.

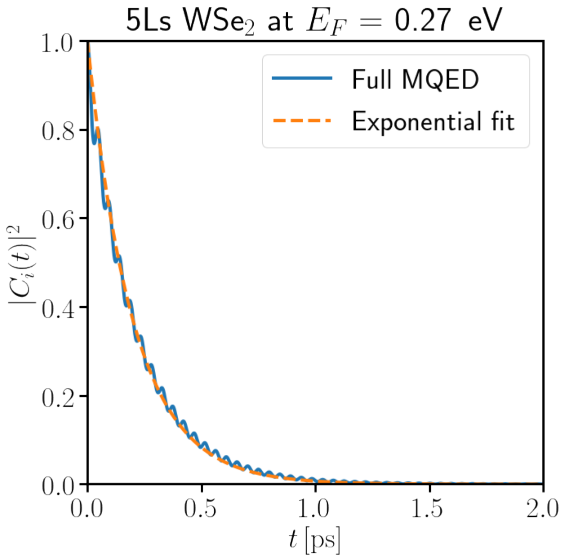

Supplementary Figure 5: Example of the time dynamics of the excited state probability for a 5 layer slab of WSe_2 at a graphene Fermi level of 0.27 eV.

hancements in the two cases. We chose these thicknesses as representative examples because they represent the systems with the largest absolute rate and largest Purcell enhancement with the lowest absolute rates respectively. From the figures we clearly see that the excited state probabilities are well decayed within the 10 ps time window used for the simulations in all cases.

Supplementary note 7: Simulation details/Convergence

As mentioned above, in order to access the time dynamics of the excited state probability we have to numerically integrate equation (5) from the main text. In order to ensure numerical stability, we employ a time spacing of $dt = 10^{-5}$ps which is orders of magnitude smaller than the fastest time scale of the dynamics. Furthermore, we propagate everything 10^6 steps out to 10ps, which is long enough to ensure that all dynamics is well decayed for all configurations considered in this work.

Another important parameter to converge is the number of grid points in the $(\mathbf{q}, \omega)$ grid on which we compute the integration kernel, $K(\boldsymbol{q}, \omega)$. Figure 6(a) shows the decay rates as a function of the number of points in a square (N, N) grid of (q, ω) points, and figure 6(b) shows the deviation from between the different grids, defined relative to the rate at a grid resolution of $N = 10000$. The example given in the figure is for a 2 layer slab since this is the system that converges slowest with respect to the number of points. As can be seen in the figure, the decay rates are converged to within less than 0.2 % at $N = 7000$ and so we use this grid resolution for the calculations in paper. Further, we note that in principle the integration over both q and ω runs from zero to infinity. However, in practice we can cut the q-integration off at the q_{hz} due to the conservation of in-plane momentum. The cut-off frequency is more arbitrary, but we find that a cut-off of $\omega_{\text{cut}} = 2\omega_{if\boldsymbol{q}=0}$ gives well converged results.

Finally, for the wave function integrals, we compute the wave function integrals on a real space grid with a 0.1 Å grid point spacing, which we find to be sufficient to obtain converged results. Because the electromagnetic excitations in this configuration carry significant momentum, we have to properly take the nonlocality of the matrix elements into account. To this end, we employ 10 q-points between $q = 0$ and $q = q_{\text{hz}}$, and use the wave functions according to their q. With these parameters, we find that the values of the wave function integrals are converged.

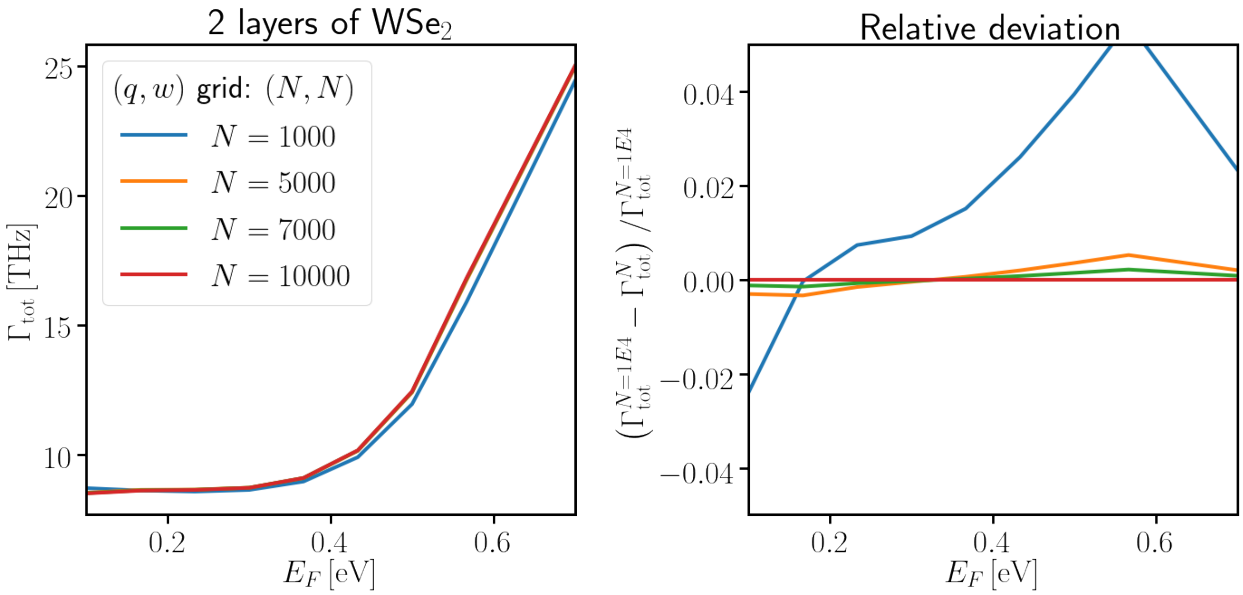

Supplementary Figure 6: This figure shows the non-pertubative decay rates calculated for a 2 layer slab of WSe_2 as a function of the grid resolution, N, of the (q, ω)-grid. These results are representative of the convergence behaviour observed across the different materials, thicknesses and graphene Fermi levels considered in this work.

References

1. Kurman, Y.; Kaminer, I. Tunable bandgap renormalization by nonlocal ultra-strong coupling in nanophotonics. *Nature Physics* **2020**, 1–7.

2. Scheel, S.; Buhmann, S. Y. Macroscopic QED-concepts and applications. *arXiv preprint arXiv:0902.3586* **2009**,

3. Novotny, L.; Hecht, B. *Principles of nano-optics*; Cambridge university press, 2012.

4. Gonçalves, P. A. D.; Peres, N. M. *An introduction to graphene plasmonics*; World Scientific, 2016.
